# Spontaneous renal artery dissection leading to multiple organ dysfunction syndrome and eventual death: A case report

**DOI:** 10.1097/MD.0000000000043230

**Published:** 2025-07-04

**Authors:** Jiahao Zhu, Yang Geng, Yingjiang Xu

**Affiliations:** a Binzhou Medical University Hospital, Binzhou City, PR China; b School of Medical Imaging (Binzhou Medical University), Binzhou City, PR China.

**Keywords:** endovascular intervention, multiple organ dysfunction syndrome, renal infarction, spontaneous renal artery dissection

## Abstract

**Rationale:**

Spontaneous renal artery dissection (SRAD) is a rare vascular emergency with high mortality. Early diagnosis remains challenging due to nonspecific clinical manifestations. This case highlights diagnostic and therapeutic dilemmas in managing SRAD, emphasizing the need for multidisciplinary collaboration.

**Patient concerns:**

A 35-year-old male with a history of left renal artery stenosis treated by percutaneous transluminal angioplasty 5 years prior presented with sudden right flank pain, nausea, and vomiting. Physical examination revealed abdominal tenderness and hypertension. Laboratory tests showed elevated homocysteine (27.8 μmol/L).

**Diagnoses:**

Contrast-enhanced computed tomography confirmed the presence of a longitudinal intimal flap in the main trunk of the right renal artery with complete distal occlusion and extensive renal infarction. No signs of aortic coarctation, mesenteric ischemia, or pulmonary embolism were found.

**Interventions:**

Emergency balloon angioplasty and stent implantation were performed. Postprocedure angiography showed partial recanalization of the main renal artery trunk but persistent distal branch occlusion.

**Outcomes:**

Despite intervention, the patient deteriorated rapidly, developing cardiac arrest, metabolic acidosis (pH 7.06, Lac 13.8 mmol/L), and multiorgan dysfunction syndrome. Resuscitation efforts failed, and the patient was declared dead 13 hours postadmission.

**Lessons:**

SRAD mimics acute abdomen or hypertensive crisis, requiring high clinical suspicion in patients with renovascular history. Endovascular intervention improved proximal flow but failed to restore distal perfusion, highlighting anatomical complexity. Chronic medial degeneration (evidenced by restenosis history) may predispose to acute dissection progression. Integration of imaging, laboratory trends, and surgical consultation is critical for timely intervention.

## 1. Introduction

Spontaneous renal artery dissection (SRAD) is a rare vascular emergency characterized by spontaneous tearing of the renal artery wall, often leading to renal ischemia, hypertension, and potential multiorgan failure. SRAD has an insidious onset and a high mortality rate (close to 25%), so early identification and intervention are urgently needed.^[1]^ Here, we report a unique case of bilateral renal arterial disease with fatal progression in which the patient experienced catastrophic progression despite timely endovascular intervention. This case underscores SRAD’s potential for rapid clinical deterioration, even in patients with known renovascular disease, and emphasizes the necessity of heightened clinical suspicion in individuals presenting with acute abdominal pain and hypertension. By integrating imaging findings, interventional outcomes, and postmortem limitations, our report provides novel insights into the intersection of chronic renal artery pathology and acute dissection, advocating for multidisciplinary management in time-sensitive emergencies.

## 2. Case presentation

**Figure 1.**
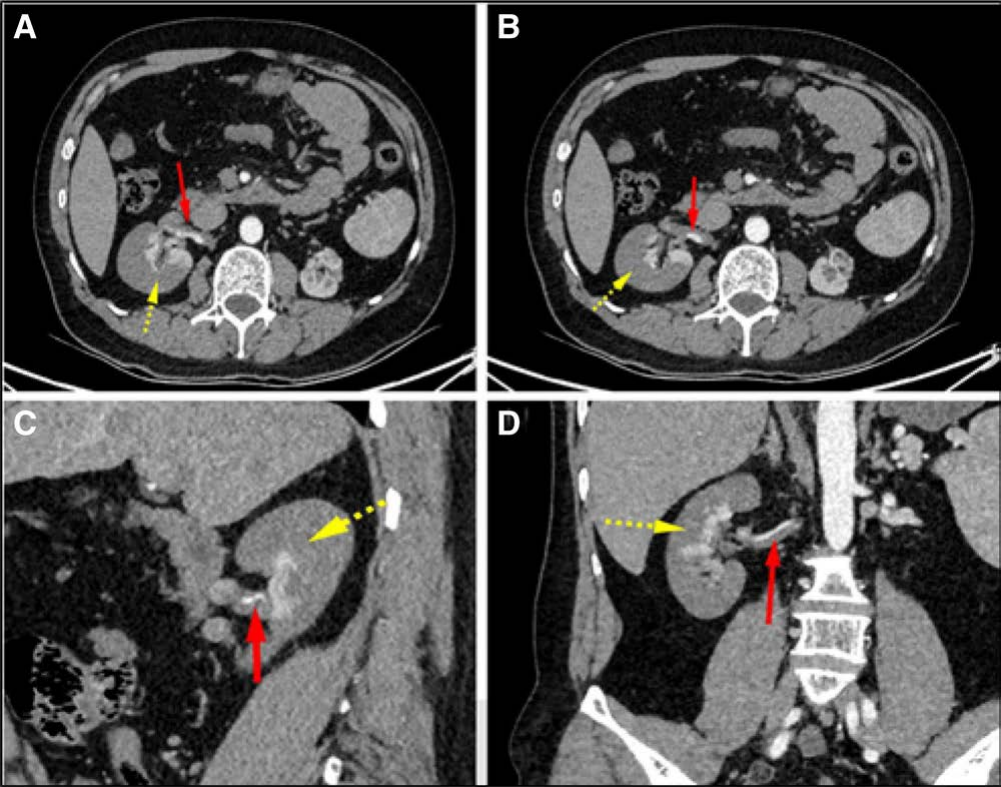
Contrast-enhanced CT scan with multiplanar reconstruction (arterial phase) revealed a linear intimal flap spanning 3.5 cm from the origin to the mid-segment of the right renal artery (Fig. 1A and B, red solid arrows), accompanied by complete absence of distal branch opacification (Fig. 1C, yellow arrows). Approximately 80% of the right renal parenchyma demonstrated wedge-shaped non-enhancement, consistent with acute total infarction (Fig. 1D, yellow dashed arrows).

The patient was a 35-year-old male with severe pain in the right side of the abdomen without any obvious cause, accompanied by nausea and vomiting, no fever, no chest tightness, and breathlessness. He was admitted to our hospital with a flat abdomen, tense abdominal muscles, and pressure pain in the whole abdomen, especially in the right lower abdomen, with no rebound pain. At 18:19 of the same day, he was admitted to the hospital for renal artery embolism with a blood pressure of 157/96 mm Hg. Five years ago, the patient presented with sudden left-sided flank pain accompanied by hypertension (systolic blood pressure 220/120 mm Hg). Computed tomography angiography (CTA) confirmed severe stenosis of the middle segment of the left renal artery (diameter reduction > 70%). Percutaneous transluminal angioplasty of the left renal artery was performed in March 2019, during which a balloon catheter was used under wire guidance to precisely dilate the stenotic segment. Immediate postoperative angiography showed improvement of the stenosis to 30%, and 24-hour ambulatory blood pressure monitoring indicated a decrease in mean systolic blood pressure to 145/85 mm Hg. The patient was prescribed regular doses of amlodipine and aspirin postoperatively, but a follow-up CTA 3 months later revealed restenosis of the left renal artery (diameter reduced by 50%), prompting a change in treatment to a combination of amlodipine and telmisartan. Subsequently, the patient’s blood pressure fluctuated between 130 and 150/80–95 mm Hg, and no further imaging studies have been conducted in the past 2 years. Laboratory tests: homocysteine was elevated (27.8 μmol/L), creatine kinase was mildly elevated (415 U/L), and D-dimer was normal (0.69 μg/mL). Contrast-enhanced CT scan of the whole abdomen and pelvis showed right renal artery embolization with right renal infarction. The CT scan revealed extensive hypoattenuation areas in the right kidney, indicating a large area of renal infarction. The dissection of the right renal artery was visualized as a linear filling defect within the vessel, extending from the origin of the renal artery towards the hilum of the kidney. The involved segment of the renal artery showed significant narrowing and irregularity (Fig. 1A–D).

**Figure 2.**
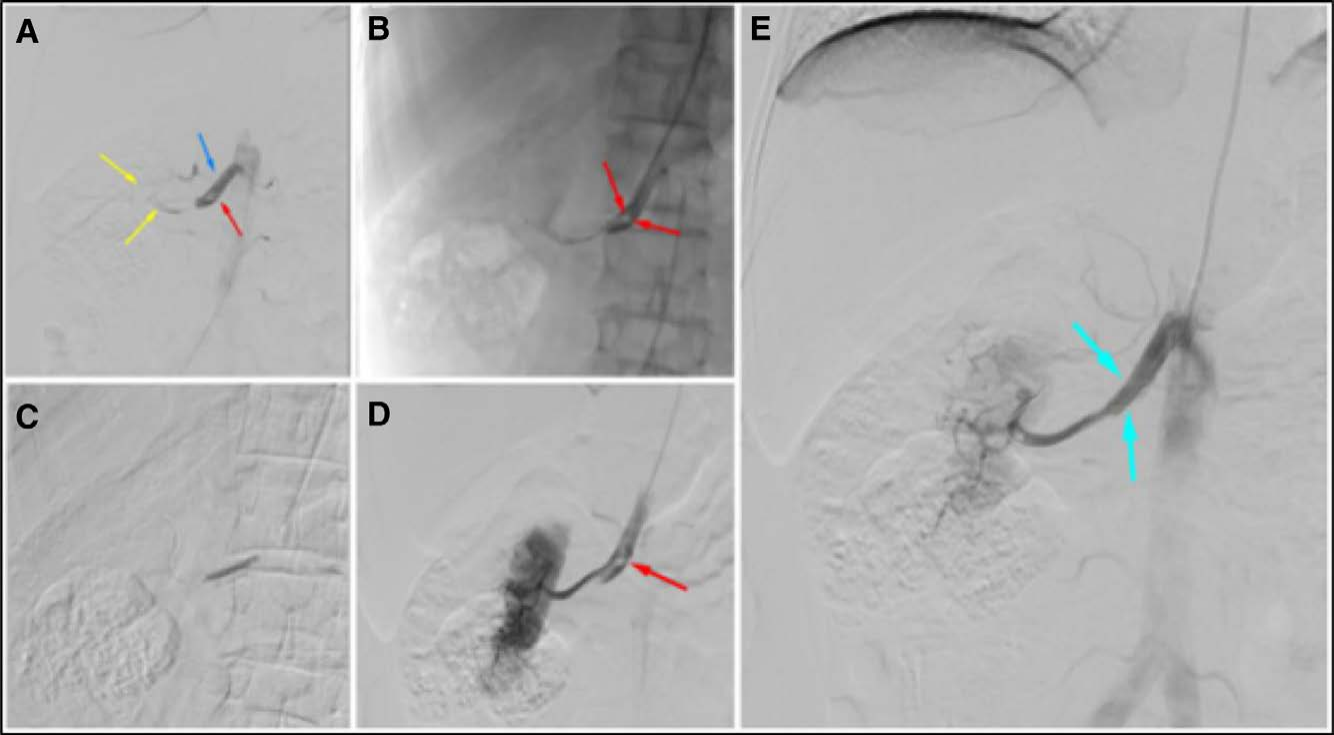
(A) The origin of the right renal artery is visualized (blue arrow), with localized signs of entrapment and filling defects (red arrow), and the distal renal artery and renal artery branches are not visualized (yellow arrows). (B) The micro-guidewire and microcatheter work together to gradually pass through the occluded segment of the renal artery, and the imaging confirms that it is located in the true lumen of the renal artery branches, with the false lumen clearly visible (red arrow). (C) A 4mm × 30mm balloon was introduced via a guidewire for dilatation. (D) After balloon dilatation, the right renal artery and some of its branches are visualized, and entrapment of the main renal artery is still visible (red arrow). (E) The SD stent was released after accurately localizing the entrapment, and reimaging demonstrated partial recanalization of the right renal artery main trunk (blue arrow), with only first-order branches visualized, indicating incomplete restoration of distal perfusion.

After consultation and discussion in our department, the risk and prevention of venous thromboembolism event were assessed using the Caprini scale, and the score was 3, with a risk level of intermediate risk. The patient underwent renal arteriography + right renal artery opening and plasty in the catheterization laboratory. During the operation, the beginning of the right renal artery could be visualized, with local entrapment and filling defects, and the distal renal arteries and renal artery branches were not visualized. The patient’s family members explained the condition of the patient and the possible regression of the patient, and the patient expressed their understanding and requested to continue the interventional therapy. Under fluoroscopic surveillance, a 4mm × 30mm balloon catheter was introduced through the V-18 guidewire, and after accurate positioning, the balloon was dilated by pressurizing and filling the balloon. Reimaging showed that the right renal artery and some of its branches could be visualized, and entrapment could still be seen in the main trunk of renal artery, and a 6mm × 18mm SD stent was introduced through the guidewire, and the stent was released successfully after accurate positioning, and the right renal artery could be visualized on reimaging (Fig. 2A–D). Post-interventional angiography demonstrated partial recanalization of the right renal artery main trunk (Fig. 2E), with only first-order branches visualized, indicating incomplete restoration of distal perfusion. At 20:14, the patient suffered from chest discomfort, followed by sudden loss of consciousness; at 21:35, the patient was transferred to the Department of Critical Care Medicine, where he was unable to breathe, his aortic pulsation had disappeared, and he was assisted in breathing by a continuous artificial respirator. Urgent blood gas analysis: PH 7.06, PO_2_ 44 mm Hg, PCO_2_ 47 mm Hg, HCO_3_^−^ 13.3 mmol/L, BE −16.9 mmol/L, Lac 13.8 mmol/L, Glu 21.6 mmol/L, Na^+^ 137 mmol/L, K^+^ 5.5 mmol/L, Ca^2+^ 1.10 mmol/L, SpO_2_ 57%. After full evaluation by the extracorporeal membrane oxygenation team of the Department of Critical Care Medicine and unanimous agreement after departmental discussion, the patient continued to have no effective cardiac activity and no arterial blood pressure during the period of extracorporeal membrane oxygenation, and the patient was declared clinically dead due to respiratory and circulatory failure and multiorgan failure at 9:40 am on the following day.

## 3. Discussion

SRAD, first described in 1944, accounts for <0.05% of all vascular dissections and remains an enigma in vascular medicine. Unlike aortic dissection, SRAD typically originates from medial degeneration without identifiable trauma or iatrogenic injury. The insidious nature of this disease stems from its variable clinical manifestations, ranging from asymptomatic progression to fatal renal ischemia. Clinical manifestations vary significantly. Contemporary literature suggests 2 distinct clinical patterns^[2]^: acute-onset flank pain with hematuria mimicking urolithiasis (60–70% cases), and “hypertensive crisis-first” presentations due to secondary renin-angiotensin-aldosterone system activation (30–40% cases). Despite advances in vascular imaging, mortality rates approach 25% due to frequent delays in diagnosis.^[3]^ This report provides novel insights into the dynamic interplay between preexisting renovascular pathology and acute dissection, through a case of bilateral renal arterial disease that progressed fatally.

Differential diagnosis: The acute presentation of flank pain and hypertension necessitated exclusion of other life-threatening conditions: Acute aortic dissection: Contrast-enhanced CT revealed no intimal tear, false lumen, or aortic dilation. Renal colic/ureteral obstruction: Absence of hydronephrosis or calculi on imaging, coupled with persistent (non-colicky) pain, argued against urolithiasis. Mesenteric ischemia: No CT evidence of bowel wall thickening, pneumatosis, or mesenteric artery occlusion. Acute pancreatitis: Serum amylase and lipase levels were within normal limits, and CT showed no pancreatic edema or necrosis. Other life-threatening conditions were also carefully considered and excluded. Acute mesenteric ischemia was ruled out based on the absence of specific abdominal signs and symptoms such as severe diarrhea and bloody stools. Pulmonary embolism was excluded by normal D-dimer levels and the absence of respiratory symptoms like dyspnea and hemoptysis. Aortic dissection was also considered, but the absence of typical symptoms such as migratory pain and the lack of findings suggestive of aortic involvement on imaging helped to exclude this diagnosis. Bedside ultrasonography showed no evidence of retroperitoneal hemorrhage or free peritoneal fluid. The combination of imaging findings, laboratory data, and clinical course strongly supported SRAD as the definitive diagnosis.

Current treatment strategies and interventions for SRAD face multiple limitations: Pharmacological therapy (e.g., antihypertensive agents, anticoagulants) is suitable for mild cases, but its efficacy is highly dependent on the patient’s condition. It is limited in effectiveness for cases with severe renal artery stenosis, aneurysms, or refractory hypertension, and cannot prevent the progression of the dissecting anatomy. It may also mask imaging features indicative of disease progression, leading to delayed intervention; Interventional therapy (e.g., stent implantation) can target stenosis, they face technical complexities – involvement of branch arteries or anatomical variations may exacerbate renal infarction, and the rate of in-stent restenosis is high (with a 5-year restenosis rate of up to 20%), requiring long-term antiplatelet therapy^[4,5]^; surgical procedures (such as renal artery reconstruction or nephrectomy) are highly invasive and pose significant challenges in postoperative management.

Numerous studies indicate that the gold standard for diagnosing renal artery entrapment is CTA followed by selective arterial angiography.^[1]^ Current imaging techniques (CTA/magnetic resonance imaging) lack sensitivity for subclinical renal ischemia and may underestimate the risk of chronic renal function deterioration. Endovascular intervention is preferred when the lesion is confined to the main stem of the renal artery. If entrapment involves the branches, stenting may worsen renal infarction. Nephrectomy is indicated when the renal artery cannot be reconstructed and the kidney is nonfunctional or completely infarcted. In the present case, despite active resuscitation and emergency vascular intervention, the patient experienced an acute onset of severe symptoms, with a history of hypertension and previous arterial stenosis surgery. Due to the extensive nature of the entrapment and insufficient restoration of blood flow, the patient developed multiple organ dysfunction syndrome and ultimately succumbed to failure to resuscitate.

What makes this case unique is that the patient had underlying bilateral renal artery lesions. Two years after percutaneous transluminal angioplasty surgery on the left renal artery, restenosis occurred, suggesting a potential structural defect in the vessel wall. Although not obtained, the pathological specimen of the right renal artery dissection is thought to show a tear in the middle elastic fibers, preventing effective relief of the persistent pressure in the false lumen. The combined effect of this chronic damage and acute events may be a key factor in the rapid deterioration of the condition.

This case redefines SRAD as a multidisciplinary time-critical emergency. The limitations of this study are primarily reflected in the following 2 aspects: First, due to the time-sensitive nature of clinical diagnostic and treatment processes, there is a certain delay between the onset of symptoms and diagnosis (specifically manifested as: nonspecific clinical symptoms at the first visit, and the attending physician did not immediately initiate vascular imaging examinations), which may affect the systematic assessment of the effectiveness of early intervention; second, due to ethical guidelines and family consent, this study did not obtain autopsy reports, resulting in the absence of pathological histological evidence (such as the extent of vascular wall structural damage and the specific pathological mechanisms underlying the origin of the dissection), which to some extent limits the in-depth analysis of the mechanisms underlying disease progression. Despite these limitations, we integrated multidimensional data from dynamic imaging studies (CTA showing the extent of dissection involvement and changes in branch perfusion; digital subtraction angiography for real-time assessment of blood flow recovery after stent placement) and laboratory indicators (dynamic monitoring of inflammatory factors and trends in renal function injury markers) to construct a diagnostic evidence chain aligned with clinical practice, providing a reference diagnostic and treatment pathway for the early identification of similar atypical renal artery dissection cases.

## 4. Conclusion

This case redefines SRAD as a multidisciplinary time-critical emergency rather than a purely vascular entity. SRAD is a rare condition with a wide range of clinical manifestations, making it prone to misdiagnosis or being overlooked. The diagnosis primarily relies on imaging tests, with early and accurate identification crucial for appropriate management. Treatment is focused on controlling blood pressure, improving blood supply to the kidney, and protecting renal function. This patient’s death underscores the severity of SRAD and highlights the importance of early intervention. It is essential that any patient presenting with abdominal pain and a relevant surgical history undergoes thorough examination, as this can aid in differential diagnosis and help prevent misdiagnosis or delayed diagnosis. At the same time, the standardized diagnosis of SRAD requires the integration of radiomics, biomarkers, and artificial intelligence technologies to establish a comprehensive system covering “early warning, precise classification, and dynamic monitoring.” This should be accompanied by the promotion of multi-center collaboration and the development of new therapies, ultimately reducing the incidence of catastrophic outcomes such as acute renal infarction and multiple organ dysfunction syndrome.

## Acknowledgments

The authors thank the patients’ families for allowing the use of patient data.

## Author contributions

**Conceptualization:** Jiahao Zhu, Yingjiang Xu.

**Data curation:** Yang Geng, Yingjiang Xu.

**Formal analysis:** Yang Geng, Yingjiang Xu.

**Funding acquisition:** Yingjiang Xu.

**Investigation:** Jiahao Zhu.

**Methodology:** Jiahao Zhu, Yang Geng.

**Resources:** Yang Geng.

**Software:** Yang Geng.

**Supervision:** Jiahao Zhu.

**Validation:** Jiahao Zhu.

**Writing – original draft:** Jiahao Zhu.

**Writing – review & editing:** Yingjiang Xu.
